# In Vitro Evaluation of Anti-Inflammatory and Protective Potential of an Extract from *Cornus mas* L. Fruit against H_2_O_2_-Induced Oxidative Stress in Human Skin Keratinocytes and Fibroblasts

**DOI:** 10.3390/ijms232213755

**Published:** 2022-11-09

**Authors:** Magdalena Wójciak, Martyna Zagórska-Dziok, Zofia Nizioł-Łukaszewska, Aleksandra Ziemlewska, Dominika Furman-Toczek, Dariusz Szczepanek, Ireneusz Sowa

**Affiliations:** 1 Department of Analytical Chemistry, Medical University of Lublin, Chodzki 4a, 20-093 Lublin, Poland; 2 Department of Technology of Cosmetic and Pharmaceutical Products, Medical College, University of Information Technology and Management in Rzeszow, Kielnarowa 386a, 36-020 Tyczyn, Poland; 3 Chair and Department of Neurosurgery and Paediatric Neurosurgery, Medical University of Lublin, 20-090 Lublin, Poland

**Keywords:** antioxidants, cornelian cherry fruit, fibroblasts, keratinocytes, oxidative stress, anti-inflammatory

## Abstract

*Cornus mas* L. is a rich source of valuable compounds with pro-health properties and, therefore, may be attractive for the pharmaceutical and cosmetic industry. This paper attempts to assess the antioxidant, anti-inflammatory, and protective effect of an extract from *C. mas* fruit on skin cells in vitro. The phytochemical analysis of the extract was carried out using UPLC-MS and the content of the main components was determined. The biological activity of the extract was assessed by in vitro analysis using two human cell lines: keratinocytes (HaCaT) and fibroblasts (BJ). Additionally, the ability of this extract to regulate gene expression (SOD-1, Nox-4) in skin cells was evaluated. Moreover, the impact of the extract and its main components, including loganic acid and cornuside, on the level of inflammatory cytokines in H_2_O_2_-treated cells was assessed. The tests showed that the extract has strong antioxidant properties and stimulates the proliferation of both types of cells. The results evidence that the *Cornus mas* L. fruit extract significantly reduces the level of reactive oxygen species in the cells tested and can modulate the expression of genes closely related to oxidative stress. Moreover, it suppresses the production of IL-6, IL-8, and TNF-α, and the effect was related to loganic acid and cornuside. The present research indicates that the analyzed dogwood extract can be an effective means of prevention of cell damage caused by free radicals and have a positive effect on the condition of skin cells.

## 1. Introduction

The importance of plant secondary metabolites is enormous. Although these compounds are not connected with basic physiological functions related to the growth and development of the plant, they have a key role in protection against pathogens and harmful environmental factors or serve as signal molecules intermediating between plant and other organisms such as plant hosts, symbiotic bacteria, or pollinators [1]. Notably, a series of pro-health properties for humans are attributed to these compounds, because they exhibit i.a. cytotoxic, anti-inflammatory, anti-bacterial, or antifungal activity [1,2,3]. Moreover, a large group of these compounds, mostly phenolics as flavonoids, phenolic acids, and anthocyanins, have strong antioxidant properties. They can regulate the oxidative-reduction processes in cells and thus support the maintenance of the redox balance [4,5]. The disturbance of the redox balance is an unfavorable process leading to the development of oxidative stress and excessive production of highly reactive oxygen species (ROS). Long-term persistence of oxidative stress may cause serious damage to cell structures as a result of the oxidation of lipids and proteins and may even lead to cell death [6]. 

The role of oxidative stress in skin aging and the development of skin-related disorders is a subject of intensive scientific research. It is known that accumulation of reactive oxygen species leads to structural and functional skin alterations, as they may modify the micro-environment of the skin and contribute to extracellular matrix (ECM) disruption [7]. Exposure to ROS leads to collagen fragmentation and disorganization of collagen fibers, which accelerates skin aging. Moreover, ROS-related cell damage leads to increased production of pro-inflammatory cytokines and, as a result, a chronic inflammatory state [8]. Therefore, the search for effective exogenous antioxidants, including natural plant substances, protecting cells against the harmful effects of free radicals via supporting the endogenous protective system is still an important task.

*Cornus mas* L. (Cornaceae), commonly known as the European cornelian cherry, is a deciduous shrub or a small tree found in nature in Central and South-Eastern Europe, and in Asia and cultivated in many countries. Its fruits (dogwood) are a rich source of valuable compounds, e.g., phenolic compounds, vitamin C, flavonoids, anthocyanins, organic acids, and iridoids, which are responsible for their pro-health properties [4,5,9,10]. Traditionally, dogwoods are applied to alleviate the symptoms of influenza and angina, relieve pain and fever, and provide astringent, hepatoprotective, and anti-inflammatory properties in gastrointestinal disorders [11]. They are the most valuable part of the plant from the point of view of pharmacy and the food industry and are often processed to produce tinctures, tonics, drinks, syrups, and jams [12]. The high content of antioxidants indicates that they can also be an attractive material for the cosmetic industry, especially given the increasing worldwide trend to use plant-based cosmetics. 

The present work attempts to evaluate the effect of an aqueous-glycerin extract from dogwood fruits on skin cells with induced oxidative stress caused by H_2_O_2_ exposure. The phytochemical composition of the extract was characterized using ESI-UPLC-MS and the main constituents were quantified. The experiments were performed on two human cell lines: keratinocytes (HaCaT) and fibroblasts (BJ). The level of intracellular reactive oxygen species (ROS) was assessed as well as the expression of genes (SOD-1, Nox-4) related to cellular stress to elucidate the protective mechanism of the examined extract on skin cells. Moreover, as oxidative stress can initiate the expression of pro-inflammatory cytokines such as tumor necrosis factor (TNF-α) and interleukin-6 (IL-6) [8], the impact of the extract on modulation of the inflammatory response in H_2_O_2_-treated cells was assessed. 

## 2. Results

### 2.1. Phytochemical Profiling of the Cornus mas L. Fruit Extract and Quantitative Analysis of the Main Constituents

The preliminary study consisted of the assessment of total phenolic (TPC), flavonoid (TFC), and anthocyanin (TAC) content. TPC, TFC, and TAC were determined from the calibration curves of gallic acid (y = 0.0046x + 0.0452, R^2^ = 0.9989), quercetin (y = 0.0153x-0.0053, R^2^ = 0.9996), and cyanidin 3-*O*-glucoside (y = 0.016x + 0.0012, R^2^ = 0.9987), respectively. The results are presented in Table 1.

**Figure 1:**
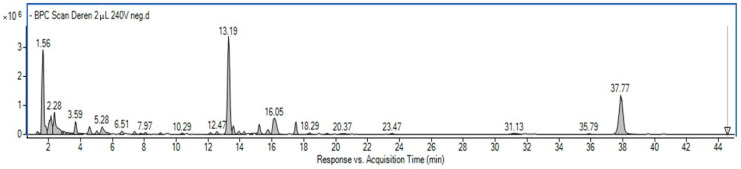
Representative BPC chromatogram of the *Cornus mas* fruit extract.

Further, the detailed composition of the glycerin-water extract from *Cornus mas* L. fruit was established using UHPLC-MS. The components were identified in the negative ionization mode based on mass data (M-H), fragmentation pattern, and UV-Vis spectra (200–600 nm) given in the literature [13,14,15,16]. Retention time, MS, and UV-Vis spectra were also compared with standards when available. A representative chromatogram is shown in Figure 1, mass data are summarized in Table 2 and the MS spectra for the main components are presented in Figure S1 (Supplementary Material).

Two groups of compounds were predominant in the analyzed extract, i.e., hydrolyzable tannins (gallotannins and ellagitannins) and iridoids. The gallotannins represented by galloyl-d-sedoheptulose, di-*O*-galloyl glucose, and two isomers of galloyl-d-glucose displayed a common MS pattern characteristic for galloyl esters with m/z 169 (gallic acid-H) and m/z 125 (decarboxylated galloyl). Two isomers of gemin D—the simplest among the ellagitannins showed the same pseudomolecular ion at m/z-H = 633.0718. The most abundant tannin compounds were galloyl-d-glucose (20.2 ± 0.25 µg/mL) and galloyl-d-sedoheptulose (31.6 ± 0.18 µg/mL). Free gallic (m/z-H 169.0151) and ellagic (m/z-H 301.0004) acids were also found in the extract, and their concentration was 34.6 ± 1.1 µg/mL and 27.4 ± 0.9 µg/mL, respectively. A low concentration of other phenolic compounds, namely protocatechuic (6.6 ± 0.3 µg/mL), chlorogenic (0.9 ± 0.1 µg/mL), caftaric (1.8 ± 0.2 µg/mL), and p-coumaroylquinic (0.5 ± 0.1 µg/mL) acids; and quercetin derivatives, rutoside (0.13 ± 0.01 µg/mL) and glucuronide (0.25 ± 0.11 µg/mL), were also found. Loganic acid, secoxyloganin, and cornuside were the main identified iridoid compounds, and loganic acid was the predominant component of the extract (224 ± 1.9 µg/mL) followed by cornuside (46.2 ± 1.1 µg/mL). Moreover, two peaks with a characteristic UV-Vis spectrum typical of anthocyanins were recorded. Based on the literature [16], they were identified as cyanidin 3-*O*-galactoside (m/z-H 447.0932 and m/z-H 285 of pseudomolecular ion corresponding to aglycone cyanidin) and pelargonidin 3-*O*-galactoside (m/z-H 431.0989 and m/z-H 269 of pseudomolecular ion corresponding to aglycone pelargonidin). Both components were also ionizable in the positive mode.

### 2.2. DPPH Radical Scavenging Assay

**Figure 2:**
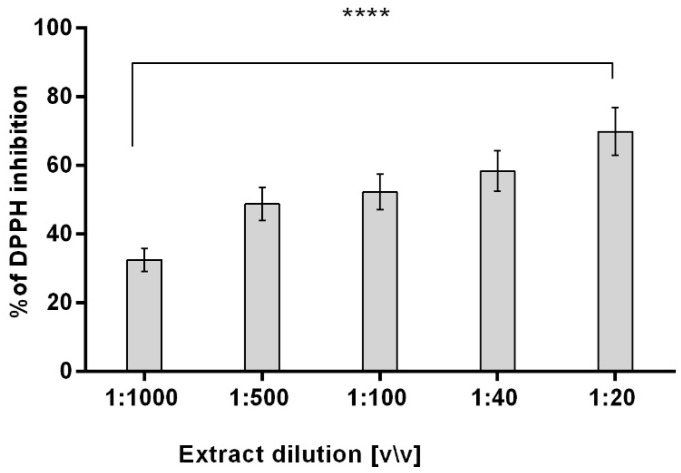
DPPH radical scavenging by various dilutions of the *Cornus mas* L. fruit extract. Data are the mean ± SD of three independent experiments, each of which consisted of three replicates per treatment group, **** *p* < 0.0001 versus the control.

The antioxidant potential was evaluated using the DPPH• scavenging assay. The results showed that the dogwood fruit aqueous/glycerin extract had significant antioxidant activity. The potency to scavenge free radicals was directly dependent on the concentration used. The lowest extract dilution (1:20 *v*\*v*) scavenged radicals almost at the level of 67% (Figure 2). The strong antioxidant potential is a result of the high polyphenolic content, especially free gallic and ellagic acids (Figure S2). In contrast, the iridoids at the tested concentration exhibited no antioxidant activity.

### 2.3. Cell Viability and Proliferation

**Figure 3:**
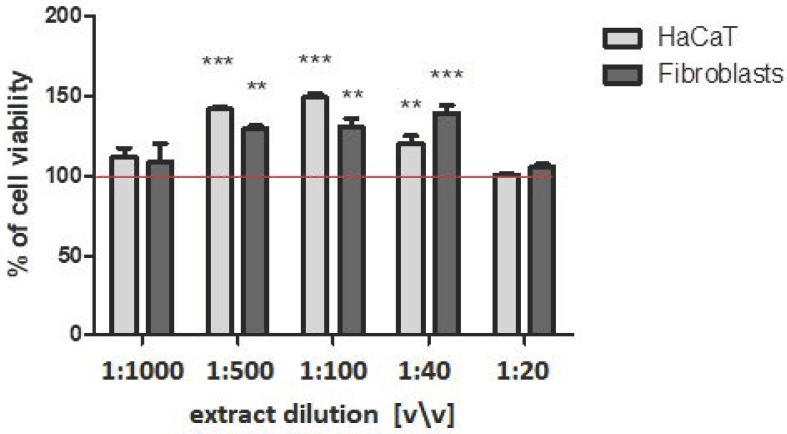
Effect of the decreasing dilutions of the aqueous/glycerin extract of *Cornus mas* L. on Neutral Red Dye uptake in cultured keratinocytes and fibroblast cells after 24 h of exposure. Data are the mean ± SD of three independent experiments, each of which consists of three replicates per treatment group. ** *p* < 0.001 versus the control (100%).

**Figure 4:**
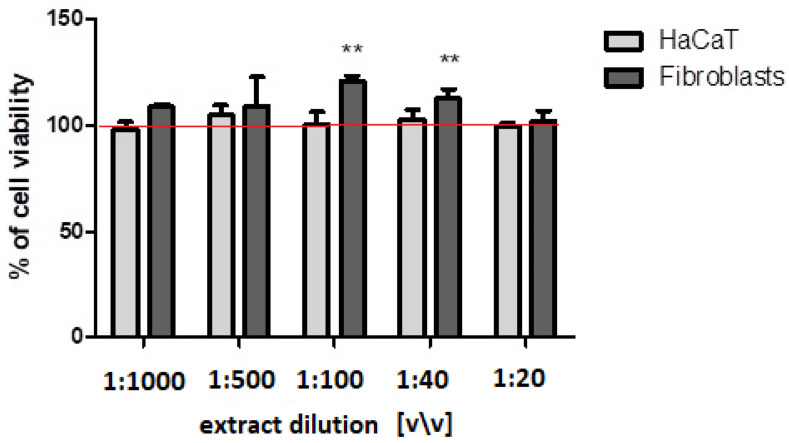
Effect of the decreasing dilutions of the aqueous/glycerin extract of *Cornus mas* L. on resazurin reduction in cultured keratinocytes and fibroblast cells after 24 h of exposure. Data are the mean ± SD of three independent experiments, each of which consists of three replicates per treatment group. ** *p* < 0.07 versus the control (100%).

The effect of the *Cornus mas* L. fruit extract on the keratinocytes and fibroblast cells was assessed using complementary tests: neutral red uptake and resazurin sodium salt reduction (Alamar Blue) assays (Figure 3 and Figure 4). It was found that, in the tested range, the extract did not negatively affect the viability of either types of cells and, therefore, showed no cytotoxicity. The viability and proliferation of the cells tested were compared to cells not treated with the extracts (the Figure 3 and Figure 4 show a red line at 100%). Moreover, at the dilution of 1:100 and 1:40 (*v*/*v*), it even stimulated fibroblasts proliferation, which was confirmed by both tests.

### 2.4. Detection of Intracellular Levels of Reactive Oxygen Species (ROS)

**Figure 5:**
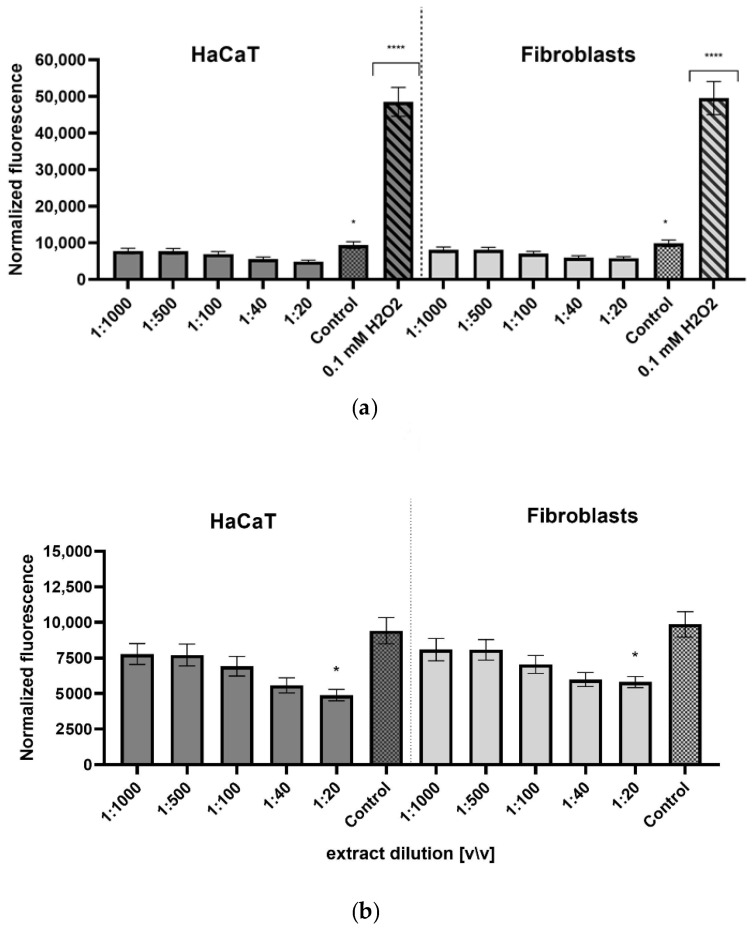
Effect of the decreasing dilutions of the aqueous/glycerin extract of *Cornus mas* L. fruit on DCF fluorescence in keratinocytes (HaCaT) and fibroblasts (BJ) with 1 mM hydrogen peroxide (H_2_O_2_) used as a positive control (**a**), and without a positive control (**b**). The data are expressed as the mean ± SD of three independent experiments, each of which consisted of three replicates per treatment group. **** *p* < 0.0001, * *p* < 0.01 versus the control.

The redox status of cells treated with the aqueous/glycerin extract was assessed using the 2’,7’-diacetate test (H_2_DCFDA) (Figure 5). The obtained results indicate that the use of *Cornus mas* L. fruit extract significantly lowered the intracellular level of reactive oxygen species in both keratinocytes and fibroblasts treated with 0.1 mM H_2_O_2_. For both types of cells treated with this compound, the increase in extract concentration was accompanied by a stronger decrease in the intracellular level of ROS, and for all the dilutions used, a lower fluorescence was achieved than in cells untreated with H_2_O_2_. These results indicate the protective antioxidant effect of the tested extract in the case of both types of cells exposed to oxidative stress caused by 0.1 mM H_2_O_2_.

### 2.5. SOD-1 and Nox-4 Expression

**Figure 6:**
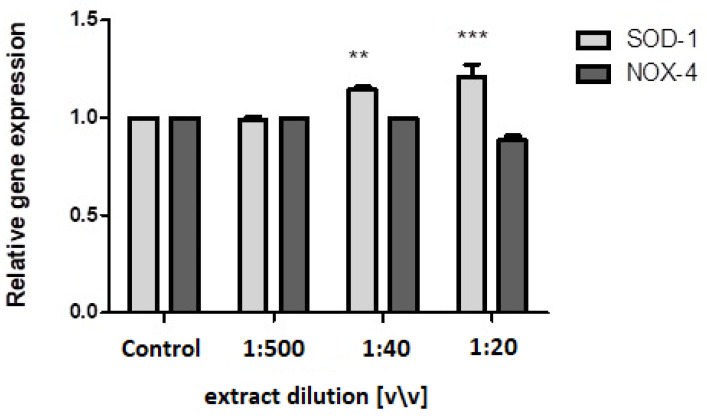
Effects of the decreasing dilutions of the aqueous/glycerin extract of *Cornus mas* L. fruit on SOD-1 and Nox-4 expression in cultured fibroblasts after 24 h of exposure. The mRNA expression was normalized to GADPH. The plot is presented in a log2 scale, where positive values indicate overexpression and negative values indicate under expression. The data are expressed as the mean ± SD of three independent experiments, each of which consisted of three replicates per treatment group. *** *p* < 0.001, ** *p* < 0.05 versus the control. 1:1000 and 1:100 dilutions had no effect on gene expression.

**Figure 7:**
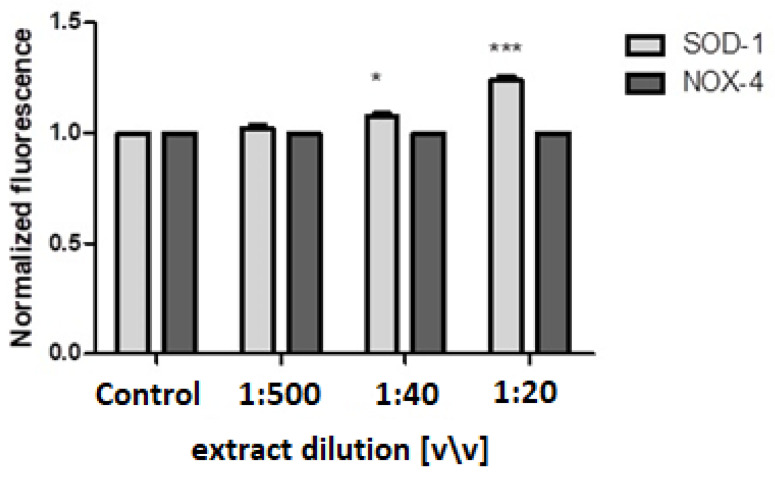
Effects of the decreasing dilutions of the aqueous/glycerin extract of *Cornus mas* L. fruit on SOD-1 and Nox-4 expression in cultured keratinocytes after 24 h of exposure. The mRNA expression was normalized to GADPH. The plot is presented in a log2 scale, where positive values indicate overexpression and negative values indicate underexpression. The data are expressed as the mean ± SD of three independent experiments, each of which consisted of three replicates per treatment group. *** *p* < 0.001, * *p* < 0.01 versus the control. 1:1000 and 1:100 dilutions had no effect on gene expression.

To evaluate the potential impact of the dogwood fruit aqueous/glycerin extract at the molecular level, we evaluated the expression of the SOD-1 and Nox-4 genes, which are known to be involved in the regulation of the oxidative stress level in cells. For this purpose, we used the PCR technique. Its results clearly indicate that the *C. mas* L. fruit extract can modulate the level of gene expression. In the fibroblast cell model, it was noticed that only the two highest concentrations upregulated the SOD-1 gene expression, whereas the Nox-4 expression was at the same level in all the concentrations tested, in comparison with the untreated cells (Figure 6). Comparable results were recorded for the HaCaT cells, in which the extract at 1:40 and 1:20 dilutions in the medium upregulated the expression of the SOD-1 gene. Additionally, in the case of HaCaT, the level of expression of the Nox-4 gene was the same in all the concentrations (Figure 7).

### 2.6. Anti-Inflammatory Assay

**Figure 8:**
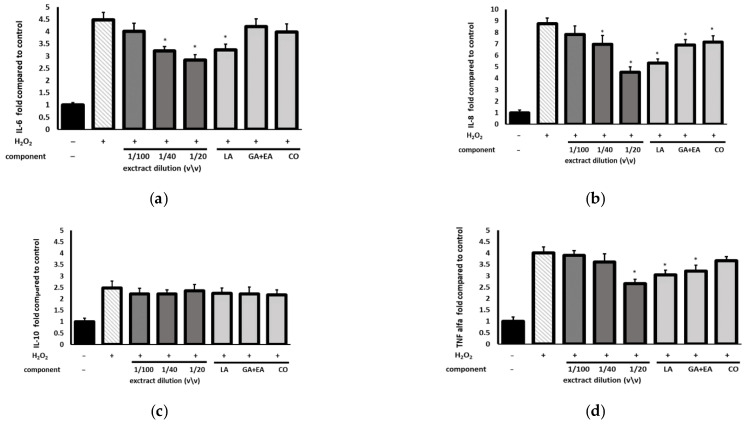
Effect of the pretreatment with the different dilutions of the *C. mas* extract or the standard compound solution prior to the H_2_O_2_ exposure on the interleukin levels calculated as a percentage in comparison with the untreated control. (**a**)—relative IL-6 level, (**b**)—relative IL-8 level, (**c**)—relative IL-10 level, (**d**)—TNF-α level. The data are means ± SD (*n* = 3). * indicates a statistically significant difference (*p* < 0.05) *versus* the H_2_O_2_-treated cells. One-way ANOVA followed by Dunnett’s multiple comparison post hoc test. LA—loganic acid at concentration of 45.0 µg/mL; GA + EA—a mixture of gallic and ellagic acid at a concentration of 7.0 and 5.5 µg/mL, respectively; CO—cornuside at a concentration of 9.2 µg/mL. The concentrations of the compounds correspond to those found in the extract at a dilution of 1:40 (*v*/*v*).

To assess the anti-inflammatory activity of the extract, the levels of proinflammatory interleukines IL-6, IL-8, tumor necrosis factor (TNF-α), and anti-inflammatory IL-10 were monitored in H_2_O_2_-treated fibroblast cells. The cells were pretreated with the *C. mas* extract or the main constituents of the extract with expected anti-inflammatory potential at concentrations corresponding to those found in the lowest extract dilution (Figure 8). Our results showed that H_2_O_2_ was a potent inductor of production of cytokines, and the level of interleukins after the H_2_O_2_ induction increased significantly (ca 2.5–8-fold). Moreover, it was found that the extract reduced the production of proinflammatory factors in a concentration-dependent manner (Figure 8a–c). Loganic acid and cornuside showed the significant anti-inflammatory activity (Figure S3) and, due to their high content in the extract, were mainly responsible for the anti-inflammatory effect. The extract and tested compounds exerted a generally minor effect on anti-inflammatory IL-10 (Figure 8c).

## 3. Discussion

Free radicals, reactive oxygen species, and reactive nitrogen species are unstable and highly reactive molecules generated continuously in the cell. Low or moderate concentrations of ROS exert beneficial effects on the functioning of the immune system; however, their excess causes numerous diseases and disorders, including skin disorders [7,8]. Their accumulation in cells generates oxidative stress, which may result in damage to numerous cell structures, lipid and protein oxidation, and changes in the DNA structure. To protect cells from ROS toxicity, several cellular antioxidant defense mechanisms are involved in the redox balance regulation. These mechanisms include such antioxidant enzymes as superoxide dismutase (SOD-1), glutathione peroxidase (GPx), and catalase (CAT) and are strongly supported by external and internal antioxidants [17,18,19]. 

*C. mas* fruit is a rich source of various antioxidants and other components with confirmed biological activity. The beneficial effect of oral administration of dogwood fruit extracts has been evidenced by various scientific groups [9,20,21]. In our study, the fruit was used to prepare the extract and a mixture of water and glycerin (80:20) was chosen as an extractant, given the potential use of the plant material in the cosmetic industry. This mixture has no cytotoxic effect on the skin. The positive effect of the extract on human skin cells was confirmed by the NR and AB assays. The former assay is based on the ability of living cells to incorporate and bind neutral red dye in lysosomes [22] and the latter is based on fluorometric evaluation of mitochondrial metabolic activity [23]. Our study showed no reduction of the neutral red dye uptake after the treatment with the *C. mas* L. extract, which indicates that the cell structures were not damaged and, therefore, the extract was nontoxic. Furthermore, the extract exerted proliferative effects in the fibroblasts and keratinocytes. However, this effect was stronger in the case of the HaCaT cells. The beneficial effect of the dogwood extract was also confirmed using the AB assay, which showed increased metabolic activity after the exposure to the extracts in both skin cell lines.

Our results showed that the obtained extract had high total phenolic content and significant antioxidant activity (DPPH assay), which is in line with other reports [2,9,20,21,24,25,26,27]. The detailed phytochemical analysis revealed i.a. a high concentration of ellagic acid as well as gallic acid and its derivatives, which are responsible for the free radical scavenging effect. The presence of hydroxyl substituents and their aromatic structure play the key role in this mechanism [28,29]. The antioxidant potential of the aqueous-glycerin dogwood extract was also confirmed in the in vitro H_2_DCFDA assay [30]. Notably, the H_2_O_2_ treatment significantly increased intracellular ROS generation by the fibroblasts and keratinocytes; however, the pretreatment with the *Cornus mas* L. fruit extract significantly decreased the ROS level in a dose-dependent manner, which confirms its strong antioxidant properties. The antioxidants present in dogwood fruits can protect lipids, proteins, and DNA against oxidative damage initiated by free radicals [31]. Our study confirmed the strong correlation between the high antioxidant activity and the content of phenolic compounds in the *Cornus mas* L. fruit extract.

The mechanism of the antioxidant activity of polyphenolic compounds may rely on direct reaction with reactive oxygen species and/or activation of antioxidant cellular systems. As shown in our study, the antioxidants from *Cornus mas* L. fruits have bidirectional activity. In addition to their strong direct ability to scavenge free radicals (DPPH test), they also modulated the expression of genes related to the antioxidant effect. Notably, the SOD-1 gene expression was upregulated at the highest concentrations of the extract. SOD is a major antioxidant enzyme that plays a crucial role in free radical scavenging [32]. In contrast, the level of expression of the Nox-4 gene remained at the same level, indicating that the tested extract was unable to modulate the expression of the Nox-4 gene in the HaCaT and fibroblast cells in vitro. The increase in the SOD-1 expression confirms the antioxidant ability of the dogwood fruits extract demonstrated previously using DPPH and H2DCFDA assays. 

Data obtained by other scientists also indicate potent antioxidant effects of *C. mas* fruits related with their impact on cellular enzyme systems. Francik et al. reported elevated catalase activity in the brain tissue of Wistar rats upon supplementation with these fruits [33]. Other studies showed a protective effect of *Cornus mas* L. fruit extracts against cisplatin-induced nephrotoxicity in Vero cell culture in vitro. The authors showed that glutathione peroxidase and superoxide dismutase activities were significantly elevated following the administration of the dogwood fruit extract [34]. Es Haghi et al. also demonstrated that treatment of rats with *C. mas* L. fruit extracts improved the level of antioxidant enzymes such as superoxide dismutase, catalase, and glutathione peroxidase and protected kidneys from oxidative stress [35]. Noteworthy, other authors highlight the fact that the antioxidant abilities differ between the dogwood genotypes [36]. 

Oxidative stress also initiates inflammatory processes, and there is strong evidence that ROS alters the release of cytokines in keratinocytes and fibroblasts [37]. In line with the research data, the present study demonstrated that H_2_O_2_ significantly increased the production of TNF-α, IL-6, IL-8, and IL-10, and their level was elevated ca. 2–5-fold compared with the control. Further shown was that the *C. mas* L. fruit extract suppressed the overexpression of TNF-α, IL-6, and IL-8 in the H_2_O_2_-induced cells; however, it had a minor impact on IL-10. Notably, the effect was mostly related to the high iridoid content, especially loganic acid, which turned out to be a strong modulator of the interleukin level. As the most abundant component of the extract, it had the greatest importance in the anti-inflammatory activity. Other reports describe the anti-inflammatory action of loganic acid. Recio et al. found that this iridoid administered topically alleviated TPA-induced ear edema in mice [38] and decreased the level of TNF-α and IL-6 in a diet-induced hypercholesterolemic rabbit model when administered orally [39]. The research conducted by Wei demonstrated an anti-inflammatory effect of iridoids, e.g., loganic acid in human neutrophils induced by inflammatory stimuli [40]. In turn, Choi et al. [41] and Kang et al. [42] described a suppressive effect of cornuside, i.e., another iridoid present in *C. mas* L., on inflammatory mediators. Moreover, the other constituents of the extract support its anti-inflammatory effect, as they act synergistically. The research data indicate that some polyphenolic compounds, e.g., flavonoids and ellagic acid, have anti-inflammatory properties [43]. Although their concentration in the tested extract was relatively low, they may have enhanced the activity of iridoids.

## 4. Materials and Methods

### 4.1. Plant Material and Extraction Procedure

Dogwood (*Cornus mas* L., cultivar Bolestraszycki) fruits were collected in August 2016 in Bolestraszyce and kindly supplied by Arboretum Bolestraszyce, Poland. The samples were dried in a hot air oven at 40 °C for two days and used for solvent extractions. The plant extract was prepared using ultrasound-assisted extraction. Next, 5 g of the plant material was placed in the beaker and extracted with 80 mL of water and 20 mL of glycerin. UAE was carried out for 40 min at room temperature. The extract was then collected and filtered through Whatman filter paper No. 10. After the preparation, the extract was stored in the dark at 4 °C for further analysis.

### 4.2. Total Phenolic Content 

The total phenolic content of the dogwood fruit extract was determined spectrophotometrically with the Folin-Ciocalteau method according to the procedure reported by Singleton et al. with some modifications [44]. Then, 300 μL of the extract solution and 1500 μL of 1:10 Folin-Ciocalteau reagent were mixed and, after 6 min in the dark, 1200 μL of sodium carbonate (7.5%) was added. After 2 h of incubation in the dark at room temperature, the absorbance at 740 nm was measured spectrophotometrically (Molecular Devices, Silicon Valley, CA, US, Multi-Mode Analysis Software). Data were expressed as gallic acid equivalents (GA)/100 g of dried plant material averaged from the three measurements.

### 4.3. Total Flavonoid Content

The total flavonoid content of the plant extract was evaluated using aluminum nitrate nonahydrate according to the procedure reported by Woisky and Salatino with modifications [45]. Next, 600 μL of the plant extract solution was mixed with 2400 μL of a mixture of 80% C_2_H_5_OH, 10% Al(NO_3_)_3_·9 H_2_O, and 1 M C_2_H_3_KO_2_. After 40 min of incubation at room temperature, the absorbance at 415 nm was measured spectrophotometrically (Molecular Devices, Silicon Valley, CA, US, Multi-Mode Analysis Software).). The total flavonoid concentration was expressed as quercetin equivalent (Qu)/100 g of dried plant material averaged from three independent measurements.

### 4.4. Total Anthocyanin Content

The total anthocyanin content was evaluated using the pH-differential method according to the procedure reported by Taghavi et al. [46]. Then, 100 µL of the extract was mixed with 300 µL of potassium chloride/hydrochloric acid buffer (0.025 M, pH 1.0) and sodium acetate/acetic acid buffer (0.4 M, pH 4.5); after 20 min, the absorbance was measured at 520 and 700 nm. TAC was calculated as o cyanidin 3-*O*-glucoside equivalent (CG)/100 g of dried plant material averaged from three independent measurements.

### 4.5. UHPLC-MS Analysis

The extract was separated using an ultra-high performance liquid chromatograph (UHPLC) Infnity Series II with a DAD detector and an Agilent 6224 ESI/TOF mass detector (Agilent Technologies, Santa Clara, CA, USA). The chromatographic conditions were as follows: an RP18 reversed-phase column Titan (Supelco, Sigma-Aldrich, Burlington, MA, USA) (10 cm × 2.1 mm i.d., 1.9 µm particle size), a thermostat temperature of 30 °C, and a flow rate of 0.2 mL/min. Water with 0.05% of formic acid (solvent A) and acetonitrile with 0.05% of formic acid (solvent B) were used as components of the mobile phase. The gradient elution program was as follows: 0–8 min from 98% A to 93% A (from 2 to 7% B), 8–15 min from 93% A to 88% A (from 7 to 12% B), 15–29 min from 88% A to 85% A (from 12 to 15% B), 29–40 min from 85% A to 80% A (from 15% to 20% B), and 40–60 min from 80% A to 65% A (from 20% B to 35% B). Chromatograms were recorded from 200 to 600 nm. The ion source operating parameters in the LC–MS analysis were as follows: drying gas temperature 325 °C, drying gas flow 8 L min^−1^, nebulizer pressure 30 psi, capillary voltage 3500 V, and skimmer 65 V. Two different values of voltage on the fragmentator were used: 180 and 240 V to obtain the mass of the components and fragment ions, respectively. Ions were acquired in the range from 100 to 1300 m/z. The MS identification was performed based on the literature data and standards. Quantification was based on calibration curves obtained using methanol standard solutions of the identified compounds. All standards, formic acid, and MS grade acetonitrile were purchased from Sigma-Aldrich (St. Louis, MO, USA).

### 4.6. DPPH Radical Scavenging Assay

The antioxidant activity of the plant extract was analyzed using the DPPH free radical scavenging assay according to the method described by Brand-Williams et al. [47]. 167 µL of 4 mM ethanol solution of DPPH was mixed with 33 µL of analyzed samples in different dilutions (1:000–1:40 *v*/*v*). The absorbance was measured at λ = 516 nm every 5 min over 30 min using a UV-Vis spectrophotometer FilterMax F5 (Thermo Fisher). A DPPH solution mixed with an equal volume of distilled water was used as the control. The percentage of the DPPH radical scavenging was calculated using the equation:

% DPPH• scavenging = [(Abs control − Abs sample)/Abs control] × 100%(1)

### 4.7. Cell Culture

HaCaT (ATCC^®^, normal human keratinocytes) and fibroblasts (ATCC^®^ CRL-2522™) were obtained from the American Type Culture Collection (Manassas, VA, USA). The HaCaT cells were maintained in DMEM (Dulbecco’s Modified Essential Medium, Gibco) with L-glutamine, supplemented with 5% (*v*/*v*) FBS (Fetal Bovine Serum, Gibco) and 1% (*v*/*v*) antibiotic (100 U/mL Penicillin and 1000 g/mL Streptomycin, Gibco). The fibroblasts were maintained in MEM (Minimum Essential Medium, Gibco) containing Earle’s salt and L-glutamine, supplemented with 5% (*v*/*v*) FBS (Fetal Bovine Serum, Gibco) and 1% (*v*/*v*) antibiotic (100 U/mL Penicillin and 1000 µg·mL^−1^ Streptomycin, Gibco). All cultured cells were kept at 37 °C in a humidified atmosphere of 95% air and 5% of carbon dioxide (CO_2_). When the cells reached confluence, the culture medium was removed from the flask (VWR) and the cells were rinsed two times with sterile PBS (Phosphate-Buffered Saline, Gibco). The confluent layer was trypsinized using 0.25% Trypsin/EDTA (Gibco) and then resuspended in fresh medium. The cells were treated with varying dilutions (1:1000, 1:500, 1:100, 1:40, 1:20 *v*/*v*) of the aqueous/glycerin dogwood fruit extract suspended in the medium. The concentration of glycerin in the samples was below 1% and did not affect the cells.

### 4.8. Cell Viability Assay

Cell growth was measured with two different methods. One was the neutral red dye (Sigma Aldrich) assay, which is based on the initial protocol described by Borenfreund et al. and determines the accumulation of the neutral red dye in the lysosomes of viable, uninjured cells [48]. The cells were placed in 96-well plates at a density of 1 × 104 cells/well with fresh medium. After 24 h of pre-culture, the medium was aspirated, and varying concentrations (0.1, 0.5, 1, 2.5, and 5%) of the dried dogwood fruit extract were added into each well and cultured for another 24 h. Unexposed cells were the control group. Following the exposure to the fruit extract, the cells were incubated for 2 h with neutral red dye (40 µg/mL) dissolved in serum free medium (DMEM or MEM for HaCaT and fibroblasts, respectively). After this, the cells were washed with Phosphate Buffered Saline (PBS), and then 150 µL of a destain solution (EtOH/AcCOOH/H_2_O_2_; 50/1/49%) was added per well, followed by gentle shaking for 10 min until the neutral red dye was extracted from the cells and formed a homogenous solution. The Neutral Red (NR) dye uptake was determined by measuring the optical density (OD) of the eluted dye at 540 nm in a microtiter plate reader spectrophotometer FilterMax F5 (Molecular Devices, Silicon Valley, CA, US, Multi-Mode Analysis Software). The other method for assessing cell viability was the resazurin sodium reduction assay called the Alamar Blue (AB) test. The assay was performed according to Page et al. [49]. The cells were placed in 96-well plates at a density of 1 × 10^4^ cells/well with fresh medium. After 24 h of pre-culture, the medium was aspirated and varying dilutions (1:1000, 1:500, 1:100, 1:40, 1:20 *v*/*v*) of the tested extract were added into each well and cultured for another 24 h. Non-treated cells maintained in the cultured medium were the control group. After the exposure, a resazurin salt solution (Sigma, R7017) was transferred into the plates to a final volume 250 µL/well and a concentration of 60 µM in medium and incubated for 3 h at 37 °C in darkness. The fluorescence was measured with an excitation wavelength of 530 nm and an emission wavelength of 590 nm using a microplate reader (FilterMax F5, Molecular Devices, Silicon Valley, CA, US, Multi-Mode Analysis Software). All experiments were performed in triplicates for each extract concentration and presented as a percentage of the control values (100%).

### 4.9. Detection of Intracellular Reactive Oxygen Species and Interleukins Level 

The cells were seeded in 96-well plates at a density of 1 × 10^4^ cells per well and initially cultured for 24 h before the experiment. After this, the culture medium was replaced with 10 µM H2DCFDA (Sigma Aldrich) in serum-free medium (DMEM or MEM for HaCaT and fibroblasts, respectively). The cells were incubated with H2DCFDA for 45 min before the treatment with the extracts. Then, the HaCaT and BJ cells were exposed to different dilutions of the extract (1:1000, 1:500, 1:100, 1:40, 1:20 *v*/*v*) for 45 min, and then treated with 1 mM hydrogen peroxide (H_2_O_2_). Cells treated with H_2_O_2_ alone were used as a positive control. Fibroblasts and HaCaT cells untreated with neither the test extract nor H_2_O_2_ were used as control cells. The DCF fluorescence was measured after every 30 min for a total 90 min using a microplate reader FilterMax F5 (Molecular Devices, Silicon Valley, CA, US, Multi-Mode Analysis Software) at maximum excitation of 485 nm and emission spectra of 530 nm. The levels of IL-6, IL-8, IL-10, and TNF-α (Elabscience, Houston, TX, USA) were measured immunoenzymatically (ELISA) using commercially available kits according to the manufacturer’s instruction. The absorbance was measured using a microplate reader at 450 nm. A stock solution of standard compounds (Sigma Aldrich) was prepared using DMSO/culture medium (1:1) and diluted to the working concentration. The final concentration of DMSO did not exceed 0.1%, and this concentration did not affect the cell viability.

### 4.10. Real-Time PCR for mRNA Expression Analyses 

For the gene expression assay, the cells were seeded on a 6-well plate at a density 5 × 10^5^ cells per well. After 24 h of pre-incubation, the cells were treated with the obtained fruit extract and incubated for 24 h. Then, the samples were collected, and total RNA was extracted from the HaCaT and fibroblast cells using a EURx Universal RNA Purification Kit according to the manufacturer’s protocol. The study was performed for all dilutions tested so far (1:1000; 1:500; 1:100; 1:40; 1:20 *v*/*v*). Test results for extract dilutions 1:1000 and 1:100 *v*/*v* not shown. The quality and quantity of the mRNA were determined spectrophotometrically at 260 and 280 nm (ND/1000 UV/VIS; Thermo Fisher NanoDrop). The reverse transcription (RT) reaction was performed at a final volume of 20 µL with 1 µg of RNA (as a cDNA template) using the High-Capacity cDNA Reverse Transcription Kit (Applied Biosystem™) according to the manufacturer’s protocol. The study was performed for all dilutions tested so far (1:1000; 1:500; 1:100; 1:40; 1:20 *v*/*v*). Test results for extract dilutions 1:1000 and 1:100 *v*/*v* not shown. The RT-PCR was run using the Bio Rad C1000 Touch™ Thermal Cycler (Bio Rad Laboratories). The products obtained from the RT reaction were amplified using the TaqMan Gene Expression Master Mix (Life Technologies Applied Biosystems, USA) kit with TaqMan probes as primes for the specific genes encoding Nox-4 (assay ID Hs00276431_m1), SOD-1 (assay ID Hs00166575_m1), and GAPDH (assay ID Hs02786624_g1). Amplification was carried out in a total volume of 20 μL containing 1× TaqMan Gene Expression Master Mix and 1 μL of the RT product, which was used as the PCR template. The standard qPCR procedures were performed as follows: 2 min at 50 °C and 10 min at 95 °C, followed by 40 cycles of 15 s at 95 °C and 1 min at 60 °C using a Bio Rad CFX Connect ™ Real-Time System (Bio Rad Laboratories). The mRNA expression was calculated relative to a nontargeting control in each experiment. The experiments were repeated in triplicate. The level of expression of the gene of interest was calculated using the comparative threshold cycle (Ct) method (Livak et al. [50] GADPH was used as the reference gene.

### 4.11. Statistical Analysis 

Each value is the mean of three replicates. The values of the different parameters were expressed as the mean ± standard deviation (SD). The two-way analysis of variance (ANOVA) and Dunnett’s posttest between groups were performed at the *p*-value of <0.05 to evaluate the significance of differences between values. Statistical analyses were performed using GraphPad Prism 8.4.3 (GraphPad Software, Inc., San Diego, CA, USA).

## 5. Conclusions

In conclusion, the present study indicates that the use of *Cornus mas* L. fruit extracts may be an effective strategy to prevent free radical-induced skin cell damage. It has demonstrated that the aqueous-glycerin extract from dried dogwood fruits is a rich source of antioxidant compounds, such as phenols and flavonoids, and is able to scavenge free radicals in a dose-dependent manner. Our analyses also show that the tested extract has a positive impact on skin cell proliferation and can reduce the intracellular ROS level. Additionally, the tested extract can modulate the expression of the SOD-1 gene, which is one of the main antioxidant enzymes providing protection against oxygen toxicity. Moreover, the study has revealed that the extract modulates the level of inflammatory agents, and the effect is mostly related to the high iridoids content. With its multidirectional activity, the extract can help in prevention of numerous skin disorders, including aging. Therefore, this extract can become a valuable raw material for the cosmetic industry. Nevertheless, further analysis should be carried out to provide more information on the mechanism of action of all compounds contained in the extract of *Cornus mas* L. 

## Figures and Tables

**Table 1:** Total phenolic, flavonoid, and anthocyanin content in the *Cornus mas* L. extract in mg per 100 g of dry weight expressed as equivalents of gallic acid (GAE), quercetin (QE), and cyanidin 3-*O*-glucoside (CG).

Total Phenolic Content (mg GA/100 g ± SD)	Total Flavonoid Content (mg QE/100 g ± SD)	Total Anthocyanin Content (mg CG/100 g ± SD)
626.0 ± 3.2	35.0 ± 1.7	47.3 ± 0.3

**Table 2:** MS data of components identified in the *Cornus mas* fruit extract in the negative ionization mode.

TR (min.)	Observed Ion Mass [M-H]-/(Fragments)	Δ ppm	Formula	Identified	Ref
1.56	191.05648	1.92	C_7_H_12_O_6_	Quinic acid	str
3.59	331.06855/(125, 169)	4.46	C_13_H_16_O_10_	O-galloyl-d-glucose ^1^	[13]
4.48	169.01506/(125)	4.78	C_7_H_6_O_5_	Gallic acid	str [13]
4.96	633.07521	2.95	C_27_H_22_O_18_	Gemin D (1)	[13]
5.28	361.07912/(125, 169)	4.10	C_14_H_18_O_11_	7-*O*-Galloyl-d-sedoheptulose ^1^	[14]
6.03	299.07843/(137)	3.96	C_13_H_16_O_8_	Hydroxybenzoic acid hexoside	[15]
7.29	633.07421	1.38	C_27_H_22_O_18_	Gemin D (2)	[13]
7.97	153.01971	2.45	C_7_H_6_O_4_	Protocatechuic acid	str, [15]
7.97	331.06801/(125, 169)	2.83	C_13_H_16_O_10_	O-galloyl-d-glucose ^1^	[13]
9.37	483.07821	0.37	C_20_H_20_O_14_	2,3-Di-galloyl glucose ^1^	[13]
13.19	375.12902	−1.37	C_16_H_24_O_10_	Loganic acid	str [16]
13.84	311.04087 (179, 135, 149)	0.05	C_13_H_12_O_9_	Caftaric acid	[15]
15.65	353.08812 (191, 179)	0.89	C_16_H_18_O_9_	Chlorogenic acid	str, [15]
16.05	491.14096 (375)	0.67	C_20_H_28_O_14_	Loganic acid derivative	[15]
17.75	447.09322 (285)	2.31	C_21_H_20_O_11_	Cyanidin 3-*O*-galactoside	[16]
18.01	431.09891 (269)	1.25	C_21_H_20_O_10_	Pelargonidin 3-*O*-galactoside	[16]
18.11	403.12490	0.78	C_17_H_24_O_11_	Secoxyloganin	[15]
19.37	337.09347 (173, 191)	1.71	C_16_H_18_O_8_	p-Coumaroylquinic acid	[15]
31.13	301.00042	4.73	C_14_H_6_O_8_	Ellagic acid	str, [13]
31.29	609.14899 (300, 463)	4.72	C_27_H_30_O_16_	Quercetin rutoside	[15]
31.81	477.06846	2.08	C_21_H_18_O_13_	Quercetin-3-*O*-glucuronide ^2^	[15]
37.77	541.15662	0.63	C_24_H_30_O_14_	Cornuside	str, [16]

Quantification was based on calibration curves for: ^1^ gallic acid, ^2^ p-coumaric acid, str—standard.

## Data Availability

The data presented in this study are available on request from the corresponding author.

## Supplementary Materials

The supporting information can be downloaded at: https://www.mdpi.com/article/10.3390/ijms232213755/s1.

## References

[1] Pagare S., Bhatia M., Tripathi N., Pagare S., Bansal Y.K. (2015). Secondary metabolites of plants and their role: Overview. Curr. Trends Biotechnol. Pharm..

[2] Gunduz K., Saracoglu O., Özgen M., Serce S. (2013). Antioxidant, physical and chemical characteristics of cornelian cherry fruits (*Cornus mas* L.) at different stages of ripenees. Acta Sci. Pol. Hortorum Cultus.

[3] Pantelidis G.E., Vasilakakis M., Manganaris G.A., Diamantidis G.R. (2007). Antioxidant capacity, phenol, anthocyanin and ascorbic acid contents in raspberries, blackberries, red currants, gooseberries and Cornelian cherries. Food Chem..

[4] Pawlowska A.M., Camangi F., Braca A. (2010). Quali-quantitative analysis of flavonoids of *Cornus mas* L. (Cornaceae) fruits. Food Chem..

[5] Seeram N.P., Nair M.G. (2002). Inhibition of lipid peroxidation and structure-activity-related studies of the dietary constituents anthocyanins, anthocyanidins, and catechins. J. Agric. Food Chem..

[6] Seeram N., Schutzki R., Chandra A., Nair M.G. (2002). Characterization, quantification and bioactivities of anthocyanins in Cornus species. J. Agric. Food Chem..

[7] Kruk J., Duchnik E. (2014). Oxidative stress and skin diseases: Possible role of physical activity. Asian Pac. J. Cancer Prev..

[8] Lee J.H., Park J., Shin D.W. (2022). The Molecular Mechanism of Polyphenols with Anti-Aging Activity in Aged Human Dermal Fibroblasts. Molecules.

[9] Yilmaz K.U., Ercisli S., Zengin Y., Sengul M., Kafkas E.Y. (2008). Preliminary characterisation of cornelian cherry (*Cornus mas* L.) genotypes for their physico-chemical properties. Food Chem..

[10] Vareed S.K., Reddy M.K., Schutzki R.E., Nair M.G. (2006). Anthocyanins in Cornus alternifolia, Cornuscontroversa, Cornuskousa and Cornusflorida fruits with health benefits. Life Sci..

[11] Kazimierski M., Regula J., Molska M. (2019). Cornelian cherry (*Cornus mas* L.)—characteristics, nutritional and pro-health properties. Acta Sci. Pol. Technol. Aliment..

[12] Szczepaniak M., Kobus-Cisowska J., Kusek W., Przeor M. (2019). Functional properties of Cornelian cherry (*Cornus mas* L.): A comprehensive review. Eur. Food Res. Technol..

[13] Przybylska D., Kucharska A.Z., Cybulska I., Sozański T., Piórecki N., Fecka I. (2020). *Cornus mas* L. Stones: A Valuable By-Product as an Ellagitannin Source with High Antioxidant Potential. Molecules.

[14] Dong Y., Feng Z.L., Chen H.B. (2018). Corni Fructus: A review of chemical constituents and pharmacological activities. Chin Med..

[15] Efenberger-Szmechtyk M., Nowak A., Czyżowska A., Kucharska A.Z., Fecka I. (2020). Composition and Antibacterial Activity of Aronia melanocarpa (Michx.) Elliot, *Cornus mas* L. and Chaenomeles superba Lindl. Leaf Extracts. Molecules.

[16] Kucharska A., Szumny A., Sokół-Łętowska A., Piórecki N., Klymenko S. (2015). Iridoids and anthocyanins in cornelian cherry (*Cornus mas* L.) cultivars. J. Food Compos. Anal..

[17] Valko M., Leibfritz D., Moncol J., Cronin M.T., Mazur M., Telser J. (2007). Free radicals and antioxidants in normal physiological functions and human disease. Int. J. Biochem. Cell Biol..

[18] Sardesai V.M. (1995). Role of antioxidants in health maintenance. Nutr. Clin. Pract..

[19] Xu D.P., Li Y., Meng X., Zhou T., Zhou Y., Zheng J., Zhang J.J., Li H.B. (2017). Natural Antioxidants in Foods and Medicinal Plants: Extraction, Assessment and Resources. Int. J. Mol. Sci..

[20] Moldovan B., Filip A., Clichici S., Suharoschi R., Bolfă P., David L. (2016). Antioxidant activity of Cornelian cherry (*Cornus mas* L.) fruits extract and the in vivo evaluation of its anti-inflammatory effects. J. Funct. Foods.

[21] Perova I.B., Zhogova A.A., Poliakova A.V., Éller K.I., Ramenskaia G.V., Samylina I.A. (2014). Biologically active substances of cornelian cherry fruits (*Cornus mas* L.). Vopr. Pitan..

[22] Repetto G., del Peso A., Zurita J.L. (2008). Neutral red uptake assay for the estimation of cell viability/cytotoxicity. Nat. Protoc..

[23] Rampersad S.N. (2012). Multiple applications of alamar blue as an indicator of metabolic function and cellular health in cell viability bioassays. Sensors.

[24] Yigit D. (2018). Antimicrobial and antioxidant evaluation of fruit extract from *Cornus mas* L. Aksaray J. Sci. Eng..

[25] Nizioł-Łukaszewska Z., Wasilewski T., Bujak T., Gaweł-Bęben K., Osika P., Czerwonka D. (2018). *Cornus mas* L. extract as a multifunctional material for manufacturing cosmetic emulsions. Chin. J. Nat. Med..

[26] Popović B.M., Stajner D., Slavko K., Sandra B. (2012). Antioxidant capacity of cornelian cherry (*Cornus mas* L.)—comparison between permanganate reducing antioxidant capacity and other antioxidant methods. Food Chem..

[27] Nizioł-Łukaszewska Z., Osika P., Wasilewski T., Bujak T. (2017). Hydrophilic Dogwood Extracts as Materials for Reducing the Skin Irritation Potential of Body Wash Cosmetics. Molecules.

[28] Villaño D., Fernández-Pachón M.S., Moyá M.L., Troncoso A.M., García-Parrilla M.C. (2007). Radical scavenging ability of polyphenolic compounds towards DPPH free radical. Talanta.

[29] Kefalas P., Kallithraka S., Parejo I., Makris D.P. (2003). A comparative study on the in vitro antiradical activity and hydroxyl free radical scavenging activity in aged red wines. Food Sci. Technol. Int..

[30] Pavelescu L.A. (2015). On reactive oxygen species measurement in living systems. J. Med. Life.

[31] Quan N.V., Xuan T.D., Tran H.D., Thuy N.T.D., Trang L.T., Huong C.T., Andriana Y., Tuyen P.T. (2019). Antioxidant, α-Amylase and α-Glucosidase Inhibitory Activities and Potential Constituents of *Canarium tramdenum* Bark. Molecules.

[32] Miao L., St Clair D.K. (2009). Regulation of Superoxide Dismutase Genes: Implications in Diseases. Free Radic. Biol. Med..

[33] Francik R., Krośniak J., Krosniak M., Berköz M., Sanocka I., Francik S. (2014). The neuroprotective effect of *Cornus mas* on brain tissue of Wistar rats. Sci. World J..

[34] Yarim G.F., Kazak F., Sozmen M., Koca I., Albayrak H., Yarim M., Cenesiz S., Ozan E. (2017). Investigation of the effect of cornelian cherry (*Cornus mas* L.) fruit extract against cisplatin-induced renal cell injury in vitro. Turk. J. Biochem..

[35] Eshaghi M., Zare S., Banihabib N., Nejati V., Farokhi F., Mikaili P. (2012). Cardioprotective effect of Cornus mas fruit extract against car-bon tetrachloride induced-cardiotoxicity in Albino rats. J. Basic Appl. Sci. Res..

[36] Ko M.J., Cheigh C.I., Chung M.S. (2014). Relationship analysis between flavonoids structure and subcritical water extraction (SWE). Food Chem..

[37] Wang Y., Sun G., Zhang Y., He J., Zheng S., Lin J. (2016). Tormentic acid inhibits H2O2-induced oxidative stress and inflammation in rat vascular smooth muscle cells via inhibition of the NF-κB signaling pathway. Mol. Med. Rep..

[38] Recio M.C., Giner R.M., Máñez S., Ríos J.L. (1994). Structural considerations on the iridoids as anti-inflammatory agents. Planta Med..

[39] Sozański T., Kucharska A., Rapak A., Szumny D., Trocha M., Merwid-Ląd A., Dzimira S., Piasecki T., Piórecki N., Magdalan J. (2016). Iridoid–loganic acid versus anthocyanins from the *Cornus mas* fruits (cornelian cherry): Common and different effects on diet-induced atherosclerosis, PPARs expression and inflammation. Atherosclerosis.

[40] Wei S., Chi H., Kodama H., Chen G. (2013). Anti-inflammatory effect of three iridoids in human neutrophils. Nat. Prod. Res..

[41] Choi Y.H., Jin G.Y., Li G.Z., Yan G.H. (2011). Cornuside suppresses lipopolysaccharide-induced inflammatory mediators by inhibiting nuclear factor-kappa B activation in RAW 264.7 macrophages. Biol. Pharm. Bull..

[42] Kang D.G., Moon M.K., Lee A.S., Kwon T.O., Kim J.S., Lee H.S. (2007). Cornuside suppresses cytokine-induced proinflammatory and adhesion molecules in the human umbilical vein endothelial cells. Biol. Pharm. Bull..

[43] Sepulveda L., Ascacio A., Rodriguez-Herrera R., Aguilera-Carbó A., Aguilar C.N. (2011). Ellagic acid: Biological properties and biotechnological development for production processes. Afr. J. Biotechnol..

[44] Singleton V.L., Orthofer R., Lamuela-Raventós R.M. (1999). Analysis of total phenols and other oxidation substrates and antioxidants by means of folin-ciocalteu reagent. Methods Enzymol..

[45] Woisky R.G., Salatino A. (1998). Analysis of propolis: Some parameters and procedures for chemical quality control. J. Apis. Res..

[46] Taghavi T., Patel H., Akande O.E., Galam D.C.A. (2022). Total Anthocyanin Content of Strawberry and the Profile Changes by Extraction Methods and Sample Processing. Foods.

[47] Brand-Williams W., Cuvelier M.E., Berset C. (1995). Use of a free radical method to evaluate antioxidant activity. Food Sci. Technol..

[48] Borenfreund E., Puerner J.A. (1985). Toxicity determined in vitro by morphological alterations and neutral red absorption. Toxicol. Lett..

[49] Page B., Page M., Noel C. (1993). A new fluorometric assay for cytotoxicity measurements in-vitro. Int. J. Oncol..

[50] Livak K.J., Schmittgen T.D. (2001). Analysis of relative gene expression data using real-time quantitative PCR and the 2(−Delta DeltaC(T)) method. Methods.

