# Supplementary material for: In Vitro Evaluation of Anti-Inflammatory and Protective Potential of an Extract from Cornus mas L. Fruit against H2O2-Induced Oxidative Stress in Human Skin Keratinocytes and Fibroblasts
Source: Int J Mol Sci. 2022 Nov 9;23(22):13755. doi: 10.3390/ijms232213755 (PMC9696105; doi:10.3390/ijms232213755)
Supplement: Supplementary file 1 [file ijms-23-13755-s001.zip › ijms-2024534-supplementary.pdf]

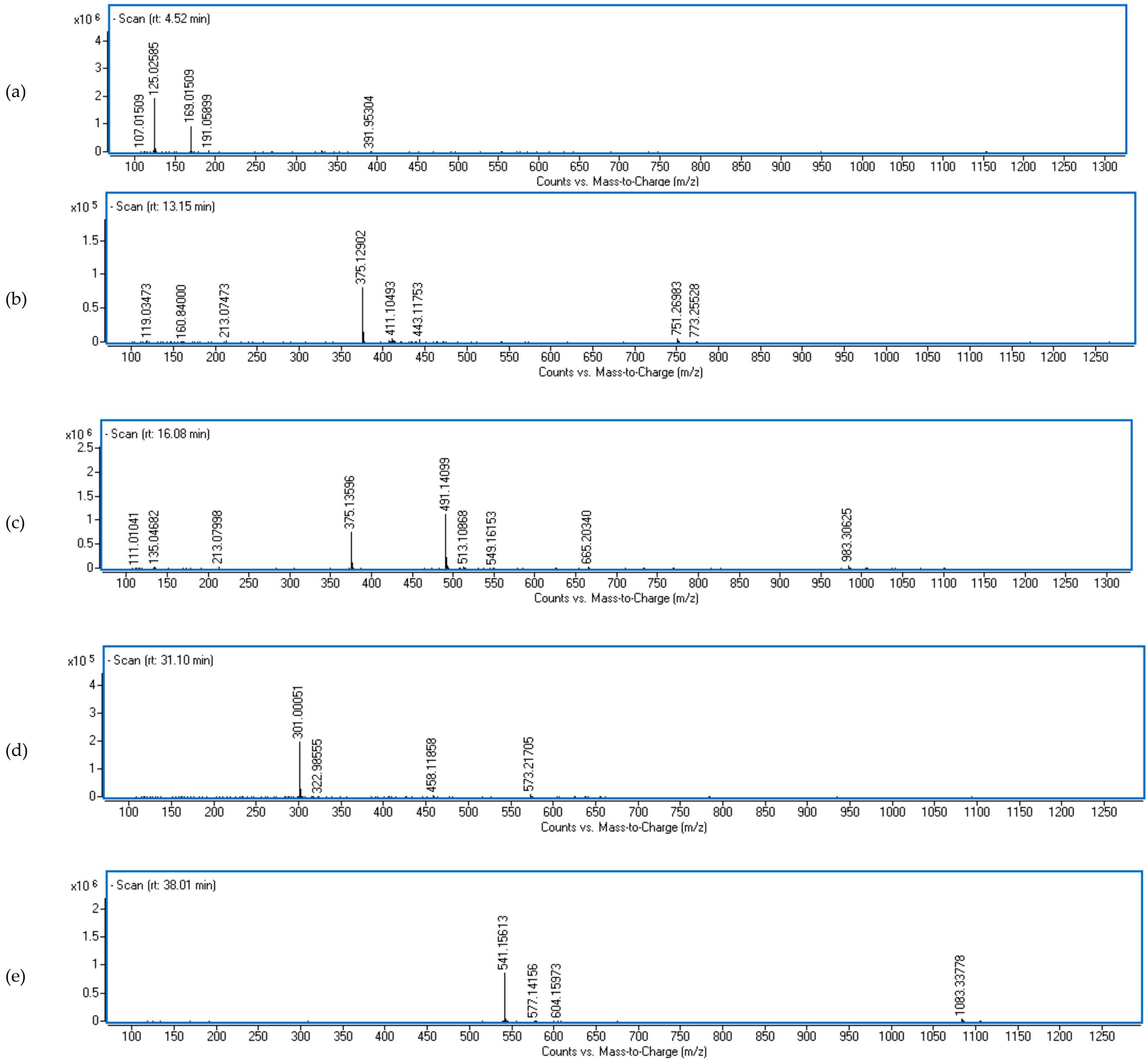

Figure S1. Representative ms spectra of main components: (a) gallic acid, (b) loganic acid, (c) loganic acid derivative, (d) ellagic acid, (e) cornuside

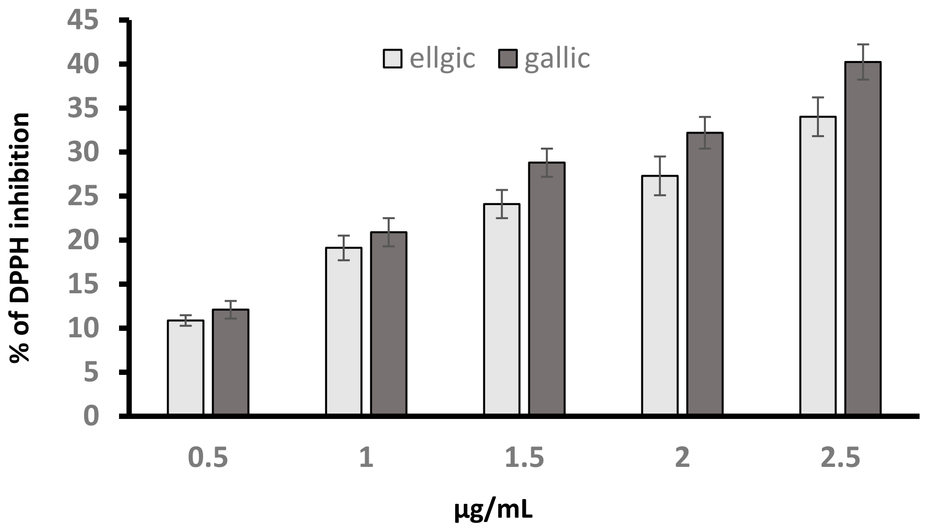

Figure S2. DPPH radical scavenging by various concentration of ellagic and gallic acid. Data are the mean ± SD of three independent experiments, each of which consisted of three replicates per treatment group.

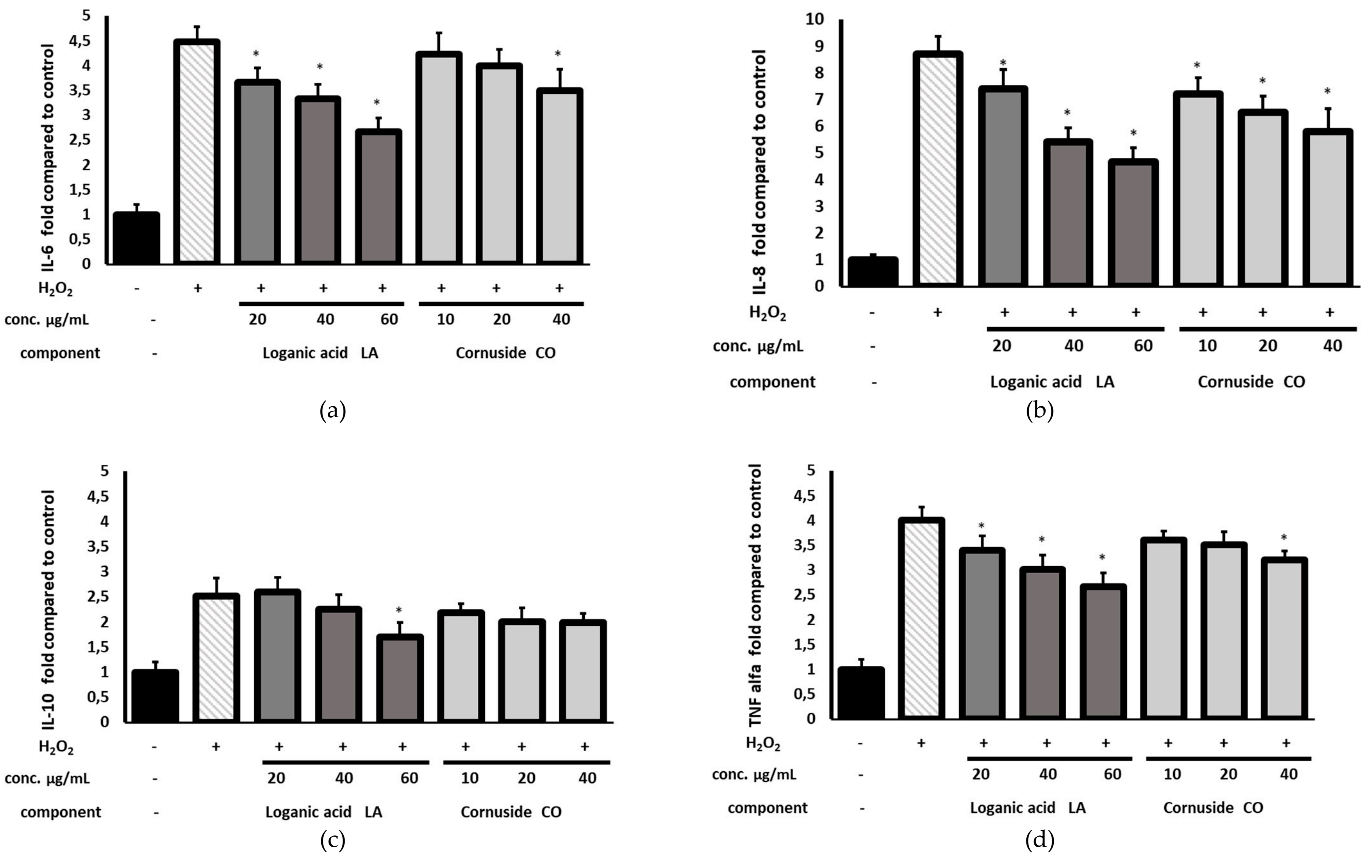

Figure S3. Effect of the pretreatment with the different concentrations of the standard compound prior to the H_2O_2 exposure on the interleukin levels calculated as a percentage in comparison with the untreated control. (a) – relative IL-6 level, (b) – relative IL-8 level, (c) – relative IL-10 level, (d) – TNF-α level. The data are means ± SD (n = 3). *indicates a statistically significant difference ($p<0.05$) *versus* the H_2O_2-treated cells. One-way ANOVA followed by Dunnett's multiple comparison post hoc test. LA-loganic acid; CO-cornuside.
